# High‐accuracy quality control method of CT system couch tops for treatment planning via an advanced 3D coordinate measuring machine

**DOI:** 10.1002/acm2.70409

**Published:** 2025-12-10

**Authors:** Ryuichi Yada, Masataka Sakamoto, Naoya Kurino, Yusuke Ueshima, Tianyuan Wang, Iori Sumida, Katsumasa Nakamura

**Affiliations:** ^1^ Department of Regional Medical Management Studies Hamamatsu University School of Medicine Hamamatsu Shizuoka Japan; ^2^ Department of Radiology Hamamatsu University School of Medicine Hamamatsu Shizuoka Japan; ^3^ Regional Creative Education Center Hamamatsu University School of Medicine Hamamatsu Shizuoka Japan; ^4^ Department of Medical Physics Kobe Proton Center Kobe Hyogo Japan; ^5^ Marketing and Clinical Solutions Accuray Japan K.K. Chiyoda‐ku Tokyo Japan; ^6^ Department of Radiation Oncology Osaka University Graduate School of Medicine Suita Osaka Japan; ^7^ Department of Radiation Oncology Hamamatsu University School of Medicine Hamamatsu Shizuoka Japan

**Keywords:** CT system, high‐precision radiation therapy, quality control method, three‐dimensional coordinate measuring machine

## Abstract

**Background:**

Quality control (QC) methods for computed tomography (CT) systems used in treatment planning have not been updated since the release of the Task Group (TG) 66 report by the American Association of Physicists in Medicine (AAPM) in 2003. Conventional QC methods for CT systems fail to fulfill the requirements of high‐precision radiation therapy. Moreover, because the geometric accuracy of CT systems can affect the accuracy of radiation therapy, which is particularly critical in high‐precision radiation therapy, a highly accurate QC method is required.

**Purpose:**

This study aimed to develop a high‐accuracy QC method for CT system couch tops suitable for high‐precision radiation therapy by utilizing a wide‐area three‐dimensional (3D) coordinate measuring machine (3D‐CMM), a type of laser tracker.

**Methods:**

We used a 3D‐CMM, which includes a wireless probe and a camera unit, focusing on a SOMATOM go.Open Pro CT system. The system was set up in accordance with the reference method outlined in the AAPM TG66 report guidelines. The initial phase verified the accuracy of 3D‐CMM measurement within a CT room using a micrometer. Subsequently, a novel continuous measurement method was developed to enable the real‐time tracking of the couch‐top displacement during travel. This new method was evaluated against the standard manual measurement method. Measurements were performed at 0.1 s intervals at various index positions on the couch top, with and without added weight to simulate the presence of a patient. The gathered data were analyzed to assess the couch‐top displacement, horizontality and orthogonality relative to the imaging plane, providing a comprehensive evaluation of the stability and alignment of the couch top.

**Results:**

The difference between the micrometer and measured shifts peaked at 0.08 mm. The continuous measurements agreed with the standard measurements within one standard deviation of the three measurements. The maximum displacements of the couch top were 5.23 and 2.00 mm in the vertical axis, with and without a weight load, respectively. There were differences in the displacement at each measurement point. In the lateral axis, the maximum displacements were 2.05 and 2.09 mm with and without a weight load, respectively. The maximum displacement in the imaging plane was observed at approximately half the distance traveled by the couch top. As the couch top traveled, the horizontal angle in the imaging plane of the couch top varied from 0.06° to 0.07° and 0.25° without a weight load and from 0.09° to 0.21° and 0.34° with a weight. The orthogonal angles of the couch top varied from 0.07° to 0.08° and 0.14° without a weight load and from 0.05° to 0.23° and 0.46° with a weight.

**Conclusions:**

The developed QC method for the couch tops of CT systems can evaluate the displacement of the couch top and its horizontality and orthogonality to the imaging plane with detailed submillimeter and subdegree accuracy levels. The high‐accuracy QC of CT systems can improve the accuracy of irradiation in radiation therapy.

## 1 INTRODUCTION

In recent years, the adoption of high‐precision radiation therapy techniques, such as intensity‐modulated radiation therapy and stereotactic irradiation (STI), has seen a significant rise.^1^, ^2^, ^3^ These techniques allow for the delivery of high radiation doses to target volumes while minimizing radiation to surrounding healthy tissues.^1^, ^2^, ^3^ High‐precision radiation therapy demands accuracy at the millimeter level or beyond to achieve the desired outcome.^4^, ^5^, ^6^, ^7^, ^8^ Consequently, this level of accuracy necessitates equivalent references of quality control (QC) in computed tomography (CT) systems used for treatment planning. It has been observed that the couch top in CT systems can experience sagging under the weight of the patient.^9^, ^10^ Deterioration in the geometric accuracy of CT systems leads to setup errors owing to differences between the CT and treatment setup. In other words, the geometric accuracy of CT systems can affect the accuracy of radiation therapy.^9^, ^11^ This problem becomes particularly critical in high‐precision radiation therapy, where the dose distribution is steep, and even minor inaccuracies can lead to significant deviations in treatment delivery.

Despite advancements in radiation therapy, the QC methods for CT systems, as outlined in the Task Group 66 (TG66) report by the American Association of Physicists in Medicine (AAPM)^11^ in 2003, have not seen significant updates in the past two decades. Consequently, these existing QC protocols fall short of fulfilling the stringent requirements of modern high‐precision radiation therapy techniques. Specifically, the reference measurement method recommended by AAPM TG66^11^ struggles to measure the couch‐top displacement with accuracy beyond the millimeter level, an accuracy that is increasingly necessary for contemporary radiation therapy practices. Additionally, the reference TG66^11^ measurement method only measures the couch‐top displacement between two measurement points. Therefore, if the couch top exhibits complex rather than linear displacements with travel, the reference TG66^11^ measurement method can result in erroneous evaluations. Given these challenges, a pressing need remains to develop a new QC measurement method for CT systems that aligns with the demands of high‐precision radiation therapy, thereby ensuring more accurate and effective irradiation treatments.

A laser tracker is an optical measuring device that determines the three‐dimensional (3D) position of a target (retroreflector) by irradiating a laser beam onto a target held in contact with the object of interest and receiving the reflected laser from the target.^12^, ^13^ A laser tracker can measure a wide area, typically several tens of meters, with an accuracy of approximately 0.01 mm.^14^ In radiation therapy, the use of laser trackers to verify the accuracy of six‐degrees‐of‐freedom (6DoF) robotic couches employed in a particle therapy system has been reported.^15^, ^16^, ^17^, ^18^ However, the conventional laser trackers used in these studies have the following two problems. First, the locations and number of targets (retroreflectors) were limited. Second, the procedure for converting the coordinate system of the laser tracker into that of the measurement object (in this study, the CT system) is complicated. Specifically, procedures are required for the geometric calibration of the camera and calculation of the transformation matrix.^15^, ^16^, ^18^

To address the limitations of conventional laser trackers used in previous studies, we explored the capabilities of a wide‐area 3D coordinate measuring machine (3D‐CMM) provided by Keyence Corporation (Osaka, Japan). A key feature of this machine is its inclusion of a probe, which provides straightforward measurements of the 3D position by positioning the probe on the desired point. The probe provides measurements even in hidden places where conventional laser trackers face difficulties owing to the limitations of the target (retroreflector) placement. Additionally, the machine measurements are traceable to the National Physical Laboratory, ensuring compliance with international standards. It boasts a minimum display resolution of 0.001 mm for distances and 0.001° for angles, with a measurement precision repeatability of ±0.01 mm, facilitating highly precise measurements.^19^

This study aimed to develop a high‐accuracy QC method for CT systems suitable for high‐precision radiation therapy by utilizing a wide‐area 3D‐CMM. In this study, the following steps were performed to establish a QC method for the couch tops of CT systems. First, using a micrometer, we demonstrated that a wide‐area 3D‐CMM can perform measurements with high accuracy in a CT room environment (see Supporting Information). Subsequently, considering measurement efficiency, we established a method for continuous measurement using a homemade jig. Upon efficiently acquiring a large amount of data, we could detect the detailed couch‐top displacement accurately, which was previously undetectable using conventional QC methods.

## 2 MATERIALS AND METHODS

### 2.1 Wide‐area 3D‐CMM

**FIGURE 1:**
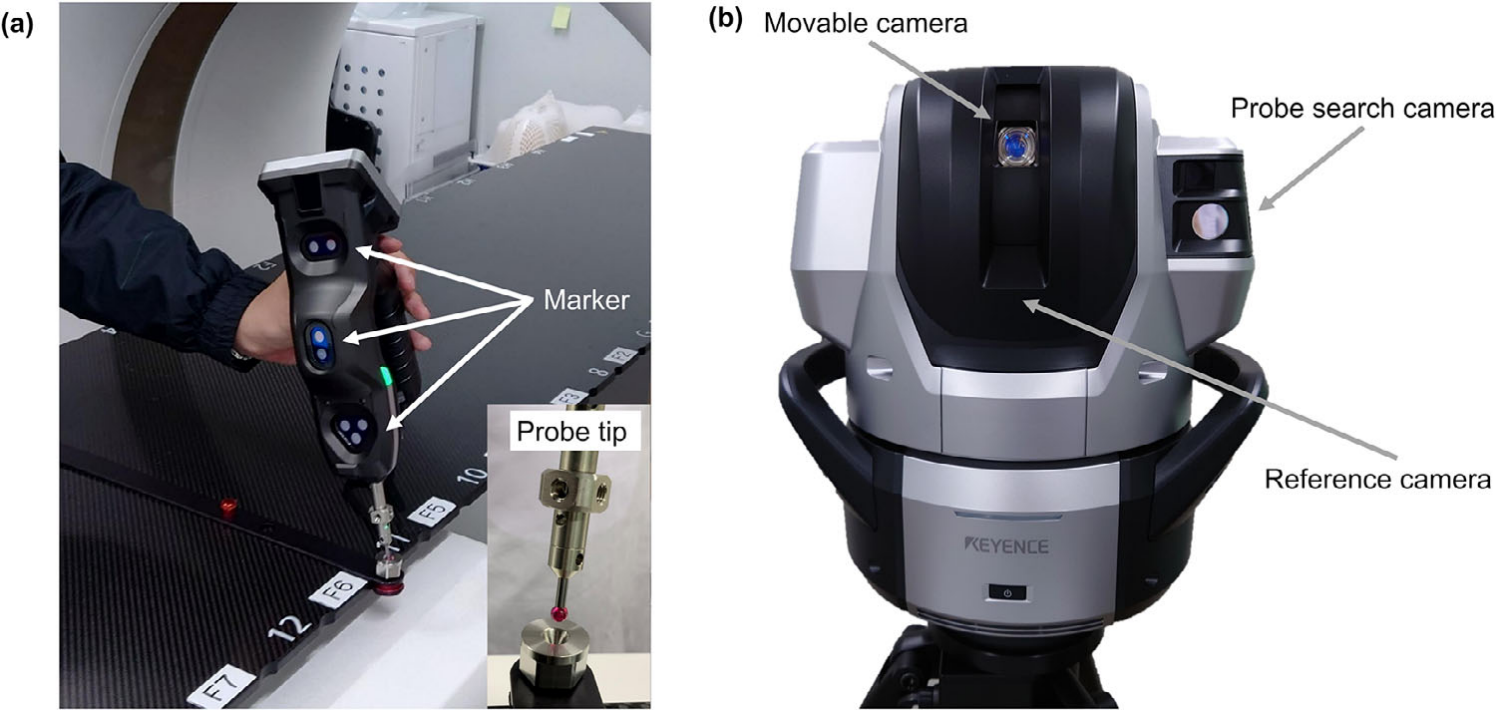
Wide‐area 3D‐CMM from Keyence Corporation. (a) Wireless probe. (b) Camera unit. Seven markers on the wireless probe emit near‐infrared light. The reference camera is not visible from the outside.

Figure 1 shows the wide‐area 3D‐CMM (WM‐3500; Keyence Co., Osaka, Japan) used in this study. The wide‐area 3D‐CMM comprises a wireless probe and camera unit. The position coordinates of the probe tip were measured based on the principle that the camera captures near‐infrared light emitted by seven markers attached to the probe. The camera unit contains three cameras. The first is a movable camera that recognizes the position and orientation of the probe while tracking its markers. The second is a probe‐search camera that constantly tracks the light emitted from the probe and instantly recognizes its position in a wide measurement area. The third is a reference camera that measures the angles of the movable camera ±90° left and right and ±30° up and down by recognizing the chart inside the camera unit.^19^ The 3D coordinates were obtained using the reference camera. The three‐camera system of this machine ensures highly accurate measurements across a broad area, boasting a repeatability of ±0.01 mm.

### 2.2 Measurement geometry

#### 2.2.1 Homemade jig and measurement point

**FIGURE 2:**
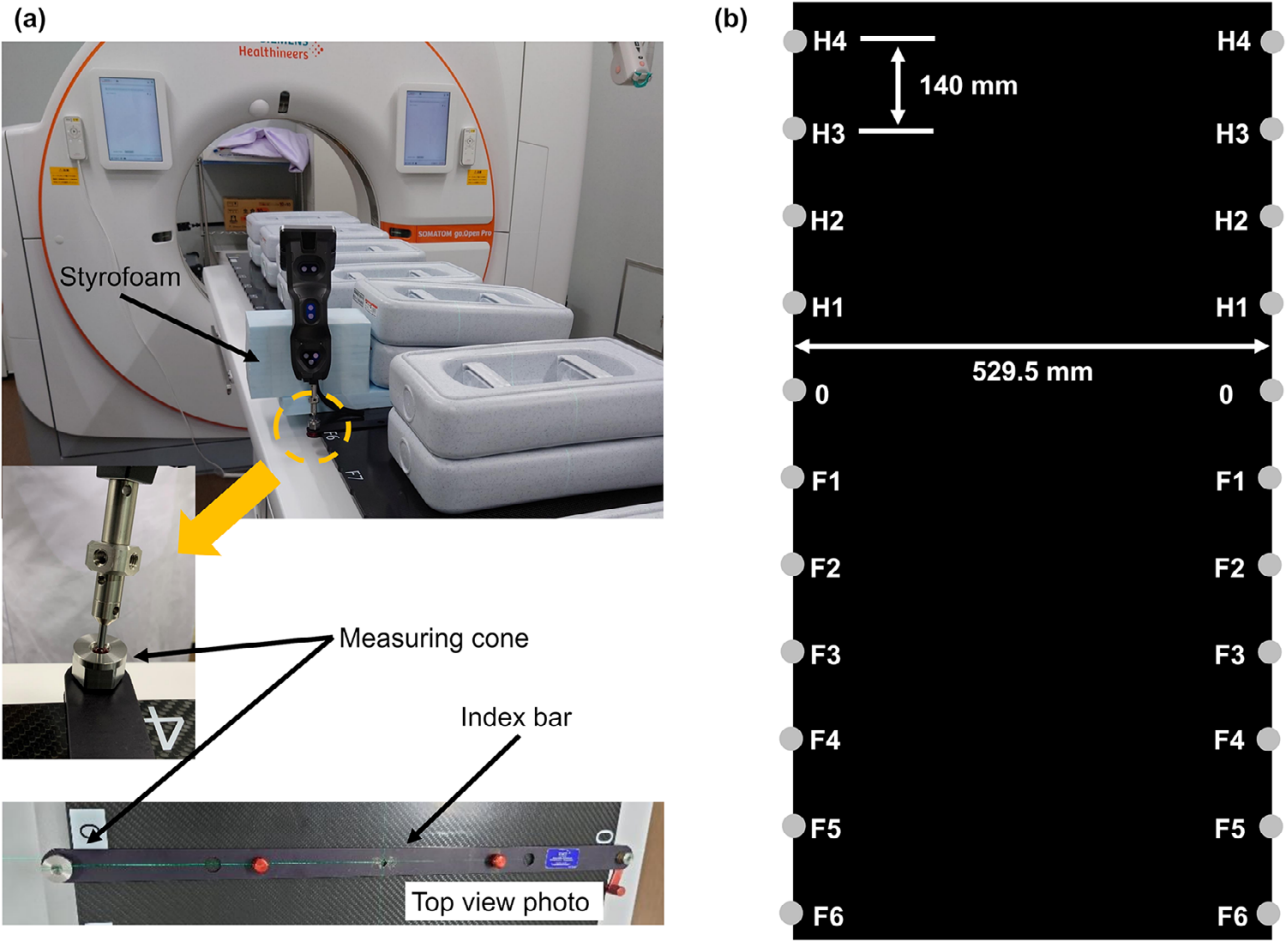
Measurement geometry. (a) Jig made from a combination of Styrofoam, a measuring cone, and an index bar. (b) Schematic top view of the computed tomography (CT) couch showing the measurement points on the CT couch. The terms H4, H3, H2, H1, 0, F1, F2, F3, F4, F5, and F6 indicate the index positions of the CT couch top. In this study, measurements were performed at 22 (11 × 2) index positions.

To obtain detailed information on the couch‐top displacement, instead of obtaining data from two points as in the reference TG66^11^ measurement method, a large amount of data was acquired at multiple measurement points. However, measuring the couch‐top displacements at multiple measurement points by repeating a series of manual probe positioning and couch‐top‐coordinate measurements with the couch stationary, followed by moving the couch top toward the gantry side (20 mm steps in this study), is time consuming. Moreover, manual positioning of the probe at the same measurement point was difficult each time. Therefore, a jig with a measuring cone attached to the index bar was created to make the probe stand on its own, which enabled us to perform continuous measurements at the same measuring point (Figure 2a). The measuring cone can be attached to both ends of the index bar to allow measurements at both ends of all index positions of the couch top. Additionally, the self‐standing position of the probe allows continuous measurement while the couch top travels.

All measurements were performed using a SOMATOM go.Open Pro CT system (Siemens Healthineers, Erlangen, Germany) installed at our institution in 2022. The measurement points for measuring the couch‐top displacements were all index positions on the SOMATOM go.Open Pro couch top where the jig could be placed (Figure 2b). Measurements were performed thrice at the same measurement points, and the standard deviation (SD) over three measurements was obtained using a spreadsheet.

#### 2.2.2 Coordinate system

**FIGURE 3:**
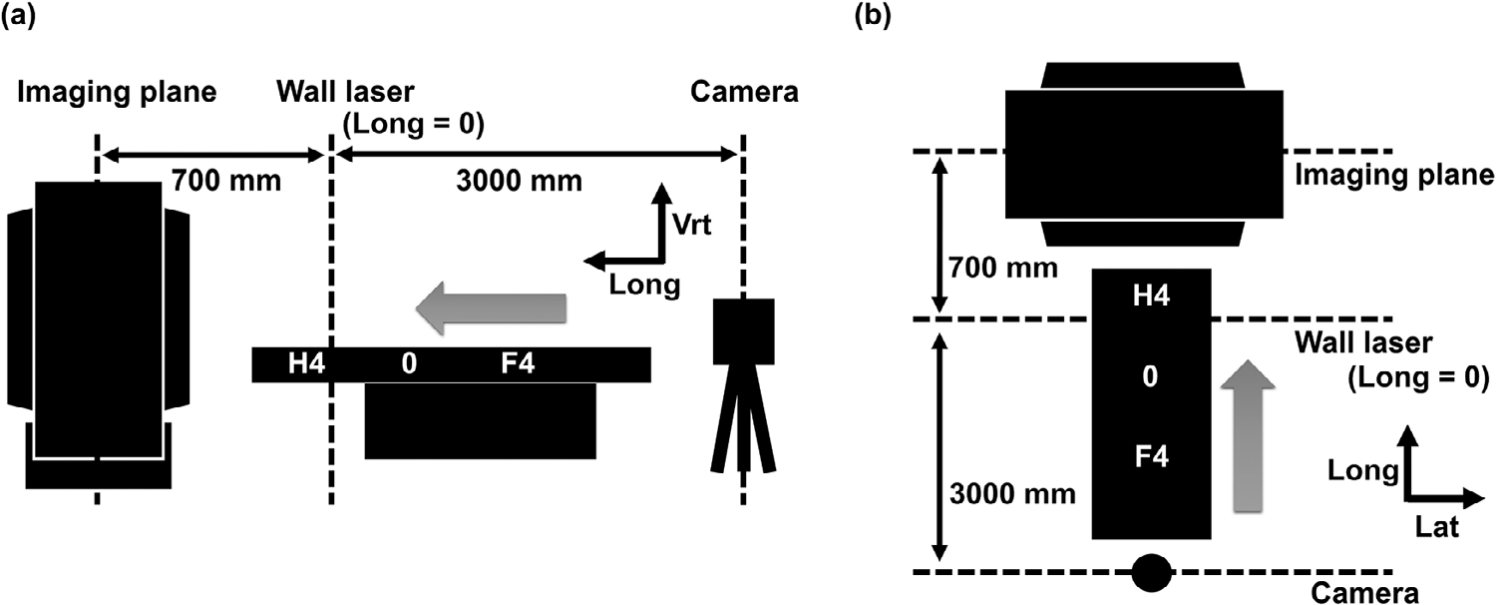
Coordinate system in the computed tomography (CT) system defined in this study. (a) Schematic side view. (b) Schematic top view. In this study, the wall laser position was set to the reference position on the longitudinal axis (Long = 0). The 3D‐CMM camera unit was located approximately 3000 mm from the wall laser position (Long = –3000).

In this study, the coordinate axes were defined for the CT system, as shown in Figure 3. The wall laser was placed 700 mm from the imaging plane and set to the reference position on the longitudinal axis (Long = 0).

To evaluate the measurement data on the defined CT system coordinate axes, the coordinate system of the wide‐area 3D‐CMM and CT system must match. Measurements were first performed with a probe on the two guide rails used to travel the couch top. The guide rail surface was set as the reference plane, and the axis parallel to the guide rails was considered the reference axis. Subsequently, two perpendicular lines were calculated using the software: one perpendicular to the reference plane and the other perpendicular to the reference axis on the reference plane. Finally, the reference axis, line perpendicular to the reference plane, and line perpendicular to the reference axis on the reference plane in the coordinate system of the 3D‐CMM were set as the longitudinal, vertical, and lateral axes in the CT coordinate system, respectively. This procedure allowed the measurement data to be evaluated directly in the coordinate system of the CT system.

### 2.3 Verification of measurement accuracy of continuous measurement method

We developed a continuous measurement method to effectively gather extensive data. The method can be used to continuously measure displacement using a homemade jig while the couch top travels. Furthermore, as the reference TG66^11^ measurement method cannot continuously measure displacement while the couch top travels, the developed continuous measurement method enables the acquisition of data that otherwise could not have been obtained. The accuracy of the 3D‐CMM relied on a standard measurement, where the probe was positioned for each measurement while the couch top was stationary. To assess the reliability of our continuous measurement method, we measured the couch‐top displacement three times using the standard and continuous measurement methods and then compared the results from each to validate the accuracy of the continuous measurement method. The measurement point was at the index position 0 on the left side of the top view of the couch top (Figure 2b) (hereafter, the left side of the couch top). The measurement points were selected randomly. In the continuous measurement method, the displacement was automatically measured every 0.1 s while the couch top traveled to the gantry side; the probe was standing on its own using the jig. In the standard measurement method, the couch top traveled to the gantry side in 20 mm steps, and measurements were performed manually each time within the same range as the continuous measurement method. The couch top was loaded with a 130 kg weight following the Japanese Industrial Standards.^20^ To distribute the weight, 12 weights of 10 kg each were placed between index positions H4 and F6, and one weight was placed on the gantry side from H4. The explicit weight distributions with clinical considerations improved the reproducibility of the weight distributions for each measurement.

### 2.4 Couch‐top displacement measurement

#### 2.4.1 Displacement per index position of couch top as couch top travels

The couch‐top displacement was measured every 0.1 s while the couch top traveled to the gantry side with the probe standing using the jig in the continuous measurement (as in Section 2.3). The measurement points were at both ends of all index positions on the couch top (Figure 2b). These 22 measurement points provided a detailed quantitative evaluation that could not be obtained using the reference TG66^11^ measurement method. The couch top traveled until the probe at each measurement point passed from the wall laser position (Long = 0) to the imaging plane (Long = 700). For index position H4 only, the couch top traveled from Long = 120 owing to the limitations of the CT system. The couch‐top displacement was measured thrice at each measurement point. The measurements were performed with and without a weight load on the couch top. The weights were loaded as described in Section 2.3.

#### 2.4.2 Couch‐top displacement in an imaging plane

In CT systems used for treatment planning, acquiring accurate images without spatial distortion is of utmost importance.^11^ The couch‐top displacement in the imaging plane must be identified when considering its effect on the image. Therefore, the position coordinates for the vertical and lateral axes in the imaging plane (Long = 700) were calculated using the data acquired as described in Section 2.4.1 by interpolation. Because continuous measurements were taken every 0.1 s—corresponding to approximately every 20 mm at a couch travel speed of 200 mm/s—the measured data were linearly interpolated at 20 mm intervals before and after the couch passed through the imaging plane.

#### 2.4.3 Displacement transition of couch‐top surface as couch top travels

The data repeatability presented in Section 2.4.1 is high to the extent that all measurement point data can be treated as acquired simultaneously. This makes it possible to understand how the couch‐top surface is displaced as the couch top travels, which has previously been difficult to evaluate quantitatively. We evaluated the displacement transition of the couch‐top surface as each index position passed through the imaging plane. The position coordinates of the probe tip at other index positions, when one index position coincided with the imaging plane, were calculated via interpolation, as described in Section 2.4.2, with an index position spacing of 140 mm.

This evaluation method enables the verification of the horizontality and orthogonality of the couch top to the imaging plane. Assuming that the couch top was rigid, the horizontality was verified from the vertical axis values at both ends of the index bar, coinciding with the imaging plane. The orthogonality was verified from the vertical axis values at the index position on the most gantry and opposite sides. The orthogonality was calculated from the average of two points in the short‐axis direction at the couch top. The angle at the couch‐top surface was calculated as an indicator.

## 3 RESULTS

### 3.1 Verification of measurement accuracy of continuous measurement method

**FIGURE 4:**
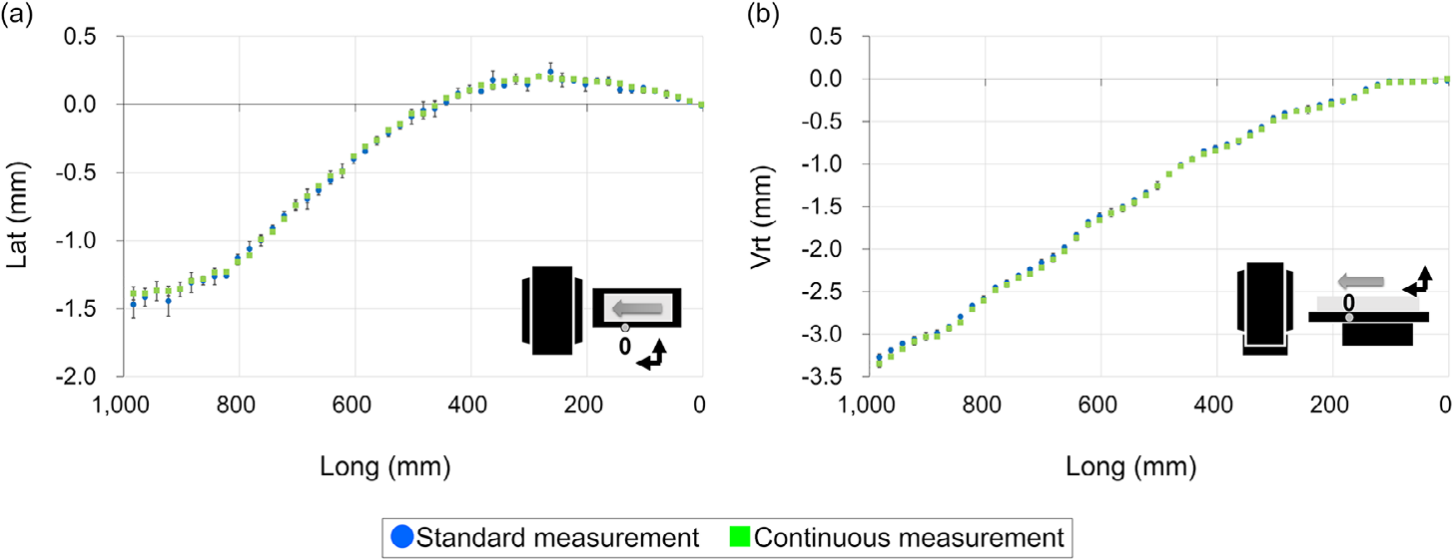
Example of the couch‐top displacement by standard and continuous measurements. The illustration in the figure represents the measurement point at a 130 kg weight load. (a) Lateral axis displacement. (b) Vertical axis displacement. Error bars are ±one standard deviation over the three measurements. The measured data were normalized by the measured values at Long = 0 for both axes.

Figure 4 illustrates the couch‐top displacement measured by the standard and continuous measurement methods. The standard measurement data, obtained at 20 mm intervals, compared with the continuous measurement data at corresponding positions along the longitudinal axis, showed that the differences were within one SD of the three measurements at all points for the lateral and vertical axes. The mean difference between the two methods across all measurement points was –0.01 ± 0.03 mm for the lateral axis and 0.02 ± 0.02 mm for the vertical axis. In terms of efficiency, each continuous measurement was completed in less than 1 min, while the standard measurement method took approximately 10 times longer.

### 3.2 Couch‐top displacement measurement

#### 3.2.1 Displacement per index position of couch top as couch top travels

**FIGURE 5:**
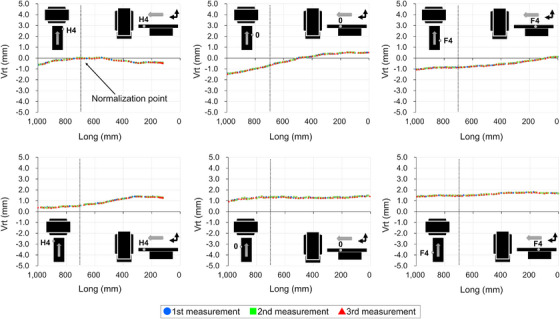
Couch‐top displacements (without weight load, vertical axis). The figure illustrates the displacement at each measurement point on the couch top, detailing the displacement on the right side (top section) and left side (bottom section) of the couch top. The data were normalized based on the measurements obtained at the right side measurement point when index position H4 was in alignment with the imaging plane (Long = 700). The dotted line within the figure signifies the location of the imaging plane.

**FIGURE 6:**
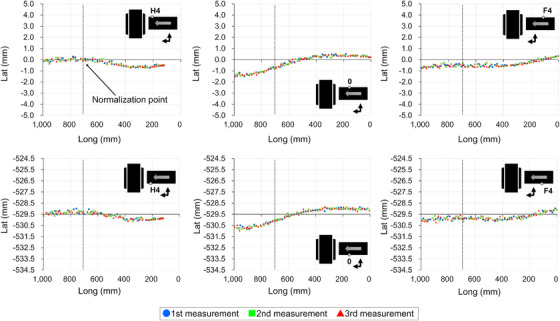
Couch‐top displacements (without weight load, lateral axis). The figure illustrates the displacement at each measurement point on the couch top, detailing the displacement on the right side (top section) and left side (bottom section) of the couch top. The data were normalized based on the measurements obtained at the right side measurement point when index position H4 was in alignment with the imaging plane (Long = 700). The dotted line within the figure signifies the location of the imaging plane.

**FIGURE 7:**
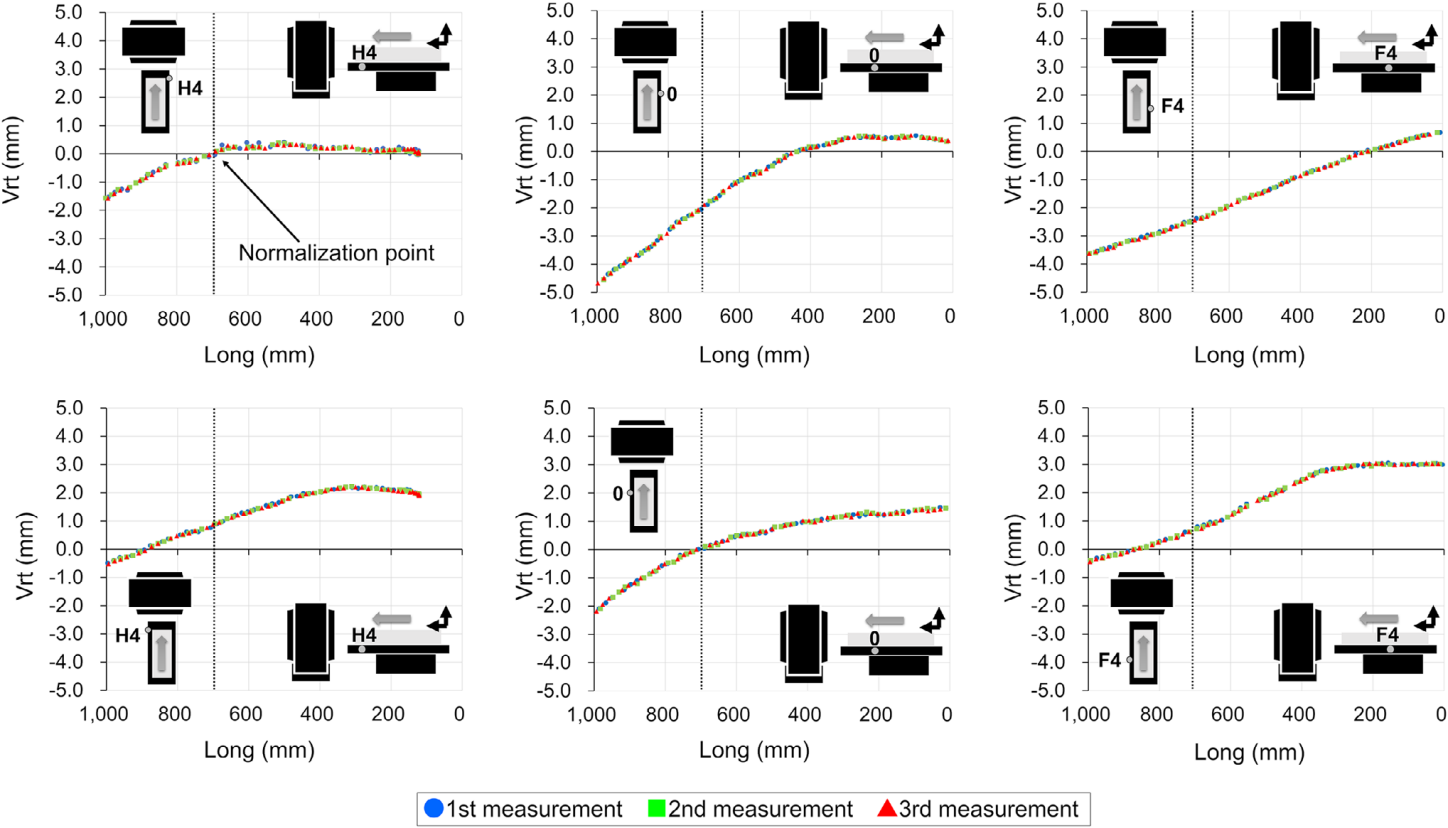
Couch‐top displacements (130 kg weight load, vertical axis). The figure illustrates the displacement at each measurement point on the couch top, detailing the displacement on the right side (top section) and left side (bottom section) of the couch top. The data were normalized based on the measurements obtained at the right side measurement point when index position H4 was in alignment with the imaging plane (Long = 700). The dotted line within the figure signifies the location of the imaging plane.

**FIGURE 8:**
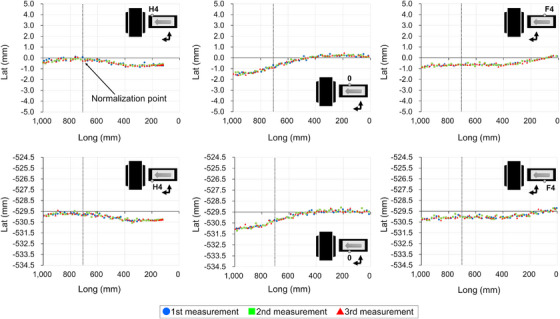
Couch‐top displacements (130 kg weight load, lateral axis). The figure illustrates the displacement at each measurement point on the couch top, detailing the displacement on the right side (top section) and left side (bottom section) of the couch top. The data were normalized based on the measurements obtained at the right side measurement point when index position H4 was in alignment with the imaging plane (Long = 700). The dotted line within the figure signifies the location of the imaging plane.

Figures 5, 6, 7, 8 show the couch‐top displacement as it traveled. Figures 5 and 6 show the couch‐top displacements without a weight load in the vertical and lateral axes, respectively. Similarly, Figures 7 and 8 show the couch‐top displacements with a weight load in the vertical and lateral axes, respectively. In this paper, the results of the couch‐top displacement measurements are presented for 6 of the 22 measurement points. All data were normalized by the measured values in the imaging plane (Long = 700) at the measurement point on index position H4 of the right side of the top view of the couch top (Figure 2b) (hereafter, the right side of the couch top). Index position H4 is the index that first passes through the imaging plane. We intentionally normalized the data at one point because normalizing the data separately at each measurement point would result in the loss of geometric relationships between the measured points.

For the vertical axis, the displacements were 5.23 mm (Figure 7) and 2.00 mm (Figure 5) with and without a weight load, respectively, as measured on the right side of the couch top at index position 0. Differences were observed in the displacement at each measurement point. This implies that differences existed in the displacement even at the two measurement points in the short‐axis direction of the couch top at the same index position. For the lateral axis, the displacements were 2.05 mm (Figure 8) and 2.09 mm (Figure 6) with and without a weight load, respectively, as measured on the left side of the couch top at index position 0. The two measurement points in the short‐axis direction of the couch top at the same index position exhibited almost the same displacement. The results with and without a weight load showed a difference in the displacement along the vertical axis, but the displacement along the lateral axis was almost the same.

Measurements were performed three times at each of the 22 measurement points with and without a weight load. A total of 132 measurements required only approximately 2 h. The maximum deviation of the three measurements at all the measurement points was 0.05 mm.

#### 3.2.2 Couch‐top displacement in an imaging plane

**FIGURE 9:**
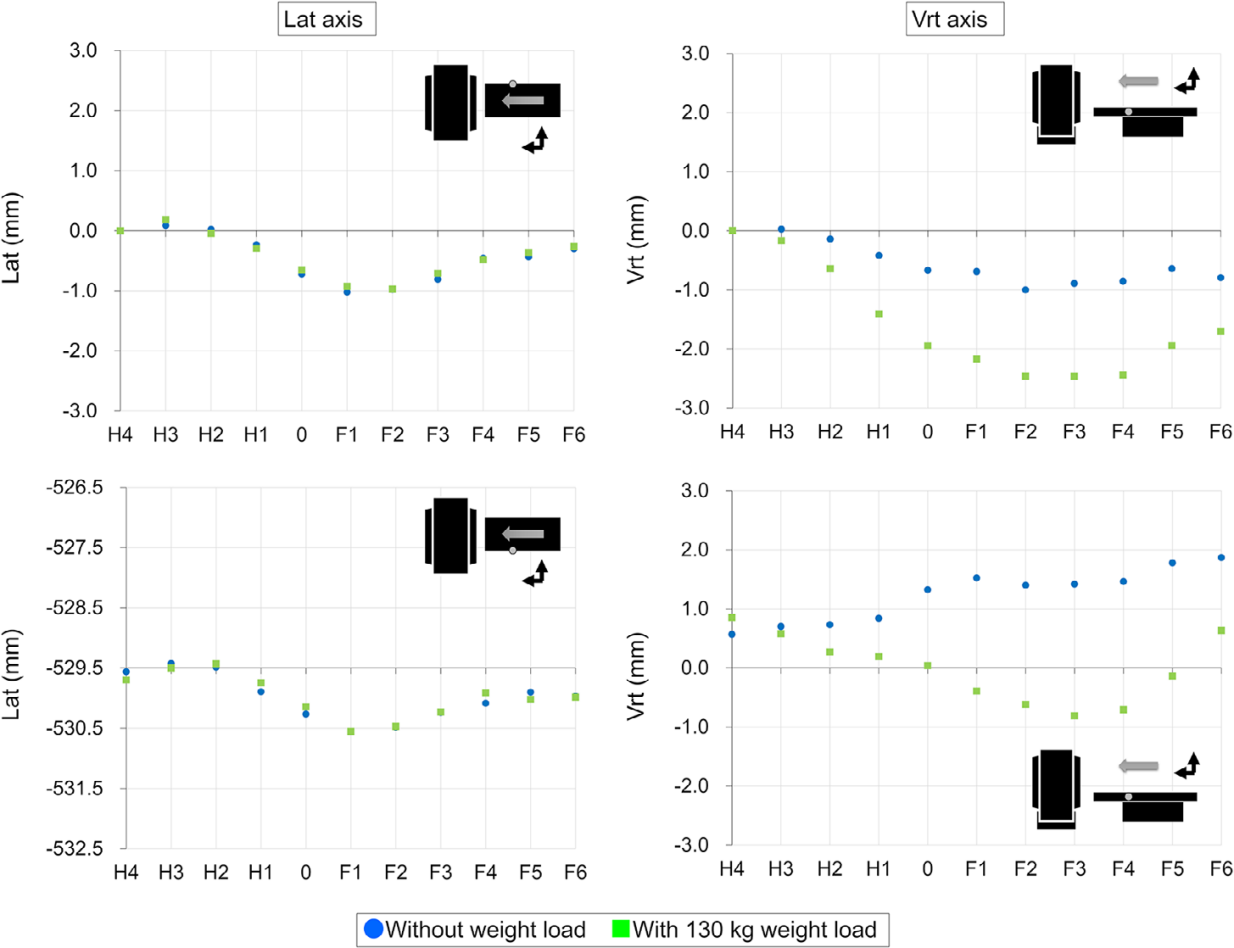
Couch‐top displacements as each index position (i.e., measurement point) coincided with the imaging plane. The displacement of the right side (top) and left side of the couch top (bottom) of the couch top are shown. The average values over three measurements are indicated. The measurement data were normalized by the measured values at the measurement point on index position H4 of the right side of the couch top, both with and without a weight load.

Figure 9 shows the couch‐top displacement when each measurement point coincides with the imaging plane. As shown in Figures 5, 6, 7, 8, the measurement data were normalized by the measured values at the measurement point on the index position H4 of the right side of the couch top, with and without a weight load.

In the lateral axis without a weight load, a maximum value of 0.96 mm was measured when the measurement point on the right side of the couch top at index position F2 coincided with the imaging plane. In the vertical axis without a weight load, a maximum value of 1.30 mm was measured when the measurement point on the left side of the couch top at index position F6 coincided with the imaged plane.

In the lateral axis with a weight load, a maximum value of 0.97 mm was measured when the measurement point on the right side of the couch top at index position F2 coincided with the imaging plane. In the vertical axis with a weight load, a maximum value of 2.47 mm was measured when the measurement point on the right side of the couch top at index position F3 coincided with the imaging plane.

With the exception of the measured data on the left side of the couch top in the vertical axis without a weight load, the maximum displacement was observed at approximately half the distance traveled by the couch top. This implies that the displacement did not increase proportionally with the couch‐top travel distance.

#### 3.2.3 Displacement transition of couch‐top surface as couch top travels

**FIGURE 10:**
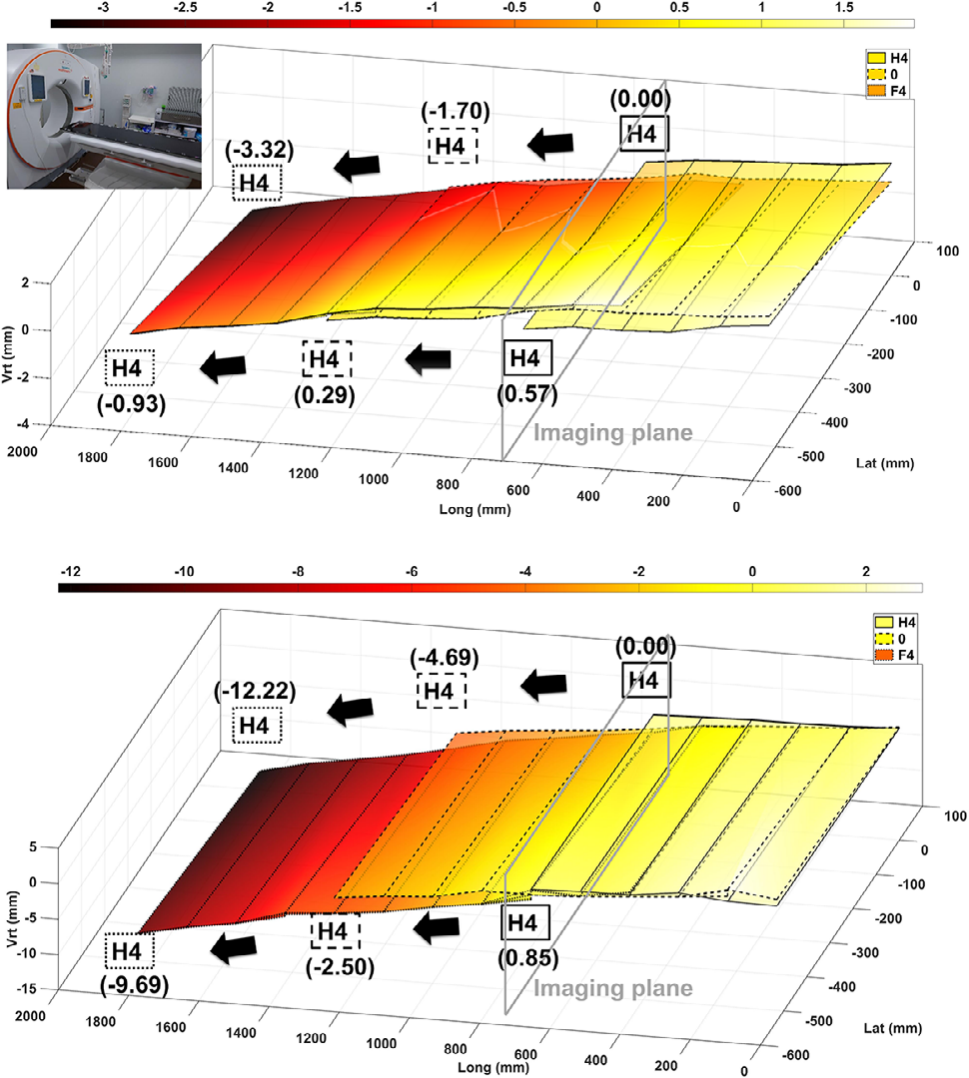
Displacement transitions of the couch‐top surface as the couch top traveled. The figure illustrates the displacements of the couch‐top surface as the couch top passes through the imaging plane sequenced by index positions H4 (represented by a solid line), 0 (depicted with a dashed line), and F4 (shown with a dotted line). The top section of the figure displays the outcomes without a weight load, whereas the bottom section depicts results under a 130 kg weight load. For instance, within the figure, the variation in the vertical axis value at the measurement point for index position H4, as influenced by the couch‐top travel, is indicated in parentheses.

The maximum deviation of the three measurements at all the measurement points acquired as described in Section 2.4.1 was 0.05 mm. Therefore, the data from all measurement points acquired separately were treated as data acquired simultaneously. Figure 10 shows the displacements of the couch‐top surface as the couch top passes through the imaging plane sequenced by index positions H4, 0, and F4. This evaluation method enabled us to visually verify the transition mechanism of the couch‐top surface as the couch top traveled. The right side of the couch top deflected more than the left side as the couch top traveled, with and without a weight load.

The horizontal angles (i.e., roll angles) in the imaging plane of the couch top without a weight load were 0.06°, 0.07°, and 0.25° at index positions H4, 0, and F4, respectively. With a weight load, the values were 0.09°, 0.21°, and 0.34°, respectively. Some differences were observed in the horizontality with and without a weight load. The orthogonal angles (i.e., pitch angles) of the couch top were 0.07°, 0.08°, and 0.14° when the index positions H4, 0, and F4 coincided with the imaging plane, respectively, without a weight load. With a weight load, the values were 0.05°, 0.23°, and 0.46°, respectively. For orthogonality, with a weight load, the couch top was confirmed to have gradually deflected as the couch top traveled.

## 4 DISCUSSION

This study aimed to develop a QC method for CT systems suitable for high‐precision radiation therapy. We developed a method for continuous measurement using a 3D‐CMM and homemade jig. The continuous measurement method efficiently and accurately acquires a large amount of data. Data from numerous measurement points revealed the complex displacement of the couch top at each position with submillimeter accuracy. Furthermore, the analysis of the large amount of acquired data showed that we could evaluate the couch‐top displacement in the imaging plane and its horizontality and orthogonality to the imaging plane in detail with submillimeter and subdegree level accuracy.

Because the measurement accuracy of 3D‐CMMs has been reported to be affected by environmental conditions such as temperature and humidity,^21^, ^22^, ^23^ the measurement accuracy of the Keyence wide‐area 3D‐CMM used in this study was first evaluated under the environmental conditions of a CT room (see Supporting Information). The difference between the shifts applied using the micrometer and the shifts measured by the 3D‐CMM peaked at 0.08 mm. Considering that the accuracy of the micrometer was 0.03 mm, the Keyence wide‐area 3D‐CMM was demonstrated to be capable of measuring with submillimeter‐level accuracy in CT rooms.

The developed continuous measurement method provides efficient and highly accurate measurements (Figure 4). The Keyence wide‐area 3D‐CMM has a probe that can easily set a coordinate system in approximately 5 min (see Section 2.2.2). However, it cannot perform continuous measurements, such as with a laser tracker. The developed continuous measurement method overcame this problem. Increased efficiency can significantly reduce the QC time.

From the data at 22 measurement points, we successfully obtained data that were not previously available and found that the couch‐top displacement differed depending on the couch‐top position (Figures 5, 6, 7, 8). Measurements can now be performed at multiple points using the developed continuous measurement method. For the QC of CT systems, the effect of the couch‐top displacement on images (i.e., evaluation in the imaging plane) must be evaluated. We evaluated this effect in detail using a large amount of data acquired with high accuracy (Figure 9). The reference TG66^11^ measurement method evaluates the couch‐top displacement in the imaging plane at two points: the head (gantry) and foot sides. The results of the measurements in this study showed the maximum displacement at approximately half the distance traveled by the couch top (Figure 9). In this case, the reference TG66^11^ measurement method underestimated the displacement. Specifically, the displacement was 2.47 mm with our measurement method but 0.22 mm with the reference TG66^11^ measurement method (calculated from measurements of the index positions H4 and F6 on the left side of the couch top). The developed measurement method can detect complex displacements with high accuracy, such as the maximum displacement at half the distance traveled by the couch top. Furthermore, the displacement data of the vertical axis on the left and right sides of the couch top enabled the detection of rolls due to couch‐top travel.

Moreover, the combination of all measured data enabled a two‐dimensional (2D) (i.e., planar) evaluation (Figure 10). The 2D evaluation enables the verification of the horizontality and orthogonality to the imaging plane of the couch top, as described in AAPM TG66.^11^ As with the couch‐top displacement, the reference TG66^11^ measurement method evaluates the horizontality and orthogonality of the couch top at two points: the gantry and foot sides. First, regarding the horizontality in the imaging plane, the developed measurement method can be evaluated at 11 points from index positions H4 to F4. This enables the detection of changes in roll as the couch top travels. Second, with respect to the orthogonality to the imaging plane of the couch top, the developed measurement method can be evaluated at an arbitrary couch‐top travel position. This implies that the orthogonality changes can be detected as the couch top travels. Additionally, the reference TG66^11^ measurement method only evaluates the displacement of the measurements in the imaging plane at two points on the gantry and foot sides of the couch top as the couch top travels. Therefore, evaluating the couch‐top surface is impossible. The developed measurement method enables the evaluation of the couch‐top surface; therefore, the orthogonal angle to the imaging plane of the couch top can be calculated. We also consider that the orthogonality should not be evaluated based on the displacement in the imaging plane. In general, the gantry side of the couch top is deflected proportionally as the couch top travels. Therefore, the orthogonality must be calculated from the coordinates of the two points on the gantry and foot sides of the couch top at the couch‐top travel position and not from the coordinates in the imaging plane.

Unlike the reference TG66^11^ measurement method, the developed measurement method did not use images for evaluation. This enables the geometric evaluation of the couch top only, as it is not affected by the image resolution, evaluation tools, or reconstruction functions owing to the image evaluation. However, because images are used for treatment planning, image‐based evaluations should be performed as required. Using a combination of both methods can be helpful for accurately identifying the causes of errors. Two points should be noted regarding the developed measurement method. First, as described in Section 2.2.2, the coordinate system was set to the couch base. Therefore, the couch base must be horizontal and orthogonal to the imaging plane. However, according to AAPM TG66,^11^ the couch base should be verified at the time of commissioning. Therefore, in the CT system used in this study, the couch base was verified at the time of couch base commissioning. Although not discussed herein, the couch base verification can be performed with high accuracy using the Keyence wide‐area 3D‐CMM. Second, in this study, the reference position of the longitudinal axis (Long = 0) was set by positioning the probe on the wall laser (see Section 2.2.2). Because of the laser thickness, a slight error occurred when the probe was positioned at the center of the laser. Therefore, the position of the imaging plane (Long = 700) is estimated to contain errors owing to errors in the positioning of the reference position. Furthermore, the wall laser accuracy was estimated as an error. However, even if an error is estimated in the definition of the imaging plane, the results in Figures 5, 6, 7, 8 clearly indicate that the error does not significantly affect the couch‐top displacement. Thus, the developed measurement method utilizing the Keyence wide‐area 3D‐CMM is considered effective as a highly accurate QC method for CT systems.

The developed QC method for CT systems sheds light on the clinical impact of CT geometric accuracy, which was previously unclear. The first aspect is the impact on treatment planning. Although the CT system installed at our institution was rigorously commissioned following the AAPM TG66^11^ measurement method, the couch‐top displacement in the vertical axis with a weight load was found to be 2.47 mm in the imaging plane (Figure 9). Displacement in the imaging plane causes image distortion, which directly affects the contouring of target volumes and organs at risk. For the CT system tested, the couch top was displaced by approximately 0.6 mm when the couch traveled 10 cm in the most displaced scanning range (from index positions H2 to 0). This value is not negligible in high‐precision radiation therapy, which demands accuracy at the millimeter level or beyond.^4^, ^5^, ^6^, ^7^, ^8^ We are currently verifying the couch‐top displacement of other CT systems, which show larger displacements, and plan to publish those results in the near future. Furthermore, the developed QC method is the first to clearly demonstrate the change in the couch‐top pitch angle (orthogonality) and roll angle (horizontality) as the couch top travels (Figures 9 and 10). Changes in these angles accompanying couch‐top travel also cause image distortion. The effect of the change in the roll angle accompanying couch‐top travel becomes more pronounced as the distance from the imaging center increases. For example, at a distance of 7 cm from the center, a rotational error of 0.5° results in a translational displacement of approximately 0.5 mm in both the vertical and lateral axes. The second aspect is the impact on image‐guided radiotherapy. If the pitch and roll angles are consistently maintained as the couch top travels, setup errors can be corrected to some extent by image registration at the time of treatment in institutions with a treatment couch capable of 6DoF correction. However, when these angles change with couch‐top travel, it is difficult to fully compensate for setup errors, even in institutions with a treatment couch capable of 6DoF correction. Several previous studies^24^, ^25^, ^26^, ^27^ have reported the effects of rotational errors on STI. A prior report proposed that the rotational error should be less than 0.5° in STI for intracranial lesions.^24^ Thus, caution is required when irradiating intracranial lesions that are separated in the craniocaudal direction using a single isocenter. Furthermore, full compensation is difficult for setup errors in craniospinal or total‐body irradiation with a long irradiation field in the craniocaudal irradiation field. This is because the tilt due to the couch‐top displacement of the CT system cannot be constant and complex (Figures 9 and 10). Moreover, these irradiations often contain two irradiation fields with two isocenters separated in the cephalocaudal direction. If image registration had been performed at each isocenter in such a treatment, the dose distribution at the junction of the two irradiation fields could have been significantly affected because of the different tilts caused by the couch‐top displacement of the CT system at each travel position. Several previous studies have reported that the dose distribution at the junction of two irradiation fields is significantly affected by setup errors,^28^, ^29^, ^30^, ^31^ which should be noted with caution.

Next, for clinical use, there are two approaches to utilize the results of the developed QC method. First, by using the data in Figures 9 and 10, CT imaging can be performed within the range where the couch‐top displacement rate is small. In other words, the position of the patient at the time of CT imaging can be adjusted, considering the imaging site. However, weight loads at the extension from the couch base reportedly affect couch‐top displacement.^10^ Therefore, additional data with more clinically relevant weight loads, considering the weight load position, rather than with equal weight loads, may be required. The second approach is to consider the couch‐top displacement when setting the planning target volume margin. A model of couch‐top displacement using a mathematical equation has been published^32^; however, the model requires extensive data within the patient weight load range to simulate clinical practice. We are currently verifying the couch‐top displacement due to different patient weights and imaging sites. Furthermore, we consider the use of the developed method at the time of commissioning to be most valuable. If commissioning could be performed with high accuracy using the developed method, the accuracy of horizontality and orthogonality to the imaging plane of the couch top could be improved. In other words, the couch‐top displacement can be reduced. For institutions with a treatment couch capable of only 4DoF correction, highly accurate commissioning of the CT system is essential. This is also true for machines such as Radixact and Halcyon, which do not have a treatment couch capable of 6DoF correction.

Given the ability of the proposed method to collect extensive data, developing efficient data analysis and evaluation software is crucial to further extend the method's functionality. Furthermore, the scope of this study was limited to the SOMATOM go.Open Pro CT system. To broaden the applicability of this method, it is essential to examine its effectiveness across various CT systems, including those from Siemens and other manufacturers. Future refinements will likely emerge from widespread implementation across multiple institutions, leveraging the portable nature of the Keyence wide‐area 3D‐CMM. Additionally, the developed measurement method holds significant potential for use in the QC of treatment couches.

## 5 CONCLUSIONS

We developed a QC method for the couch tops of CT systems by utilizing the Keyence wide‐area 3D‐CMM. The developed method accurately assesses the displacement, horizontality, and orthogonality of the couch top relative to the imaging plane, achieving submillimeter and subdegree accuracy. Such high‐accuracy QC can enhance the accuracy of irradiation in radiation therapy. Implementing this method at the time of the commissioning of CT systems could significantly improve radiation therapy accuracy. Future advancements should focus on developing a dedicated jig and software to streamline and refine the QC process, making it more straightforward and accurate.

## AUTHOR CONTRIBUTIONS

Ryuichi Yada designed the study. Ryuichi Yada, Masataka Sakamoto, Naoya Kurino, and Yusuke Ueshima performed the measurements. Ryuichi Yada and Tianyuan Wang analyzed the data. Ryuichi Yada wrote the paper and prepared figures and tables. Iori Sumida gave valuable advice on interpreting the results. Tianyuan Wang, Iori Sumida, and Katsumasa Nakamura revised the manuscript. Katsumasa Nakamura supervised the study. All authors read and approved the final manuscript.

## CONFLICT OF INTEREST STATEMENT

The authors declare no conflict of interest.

## Supporting information



Supporting information
