# Supplementary material for: High‐accuracy quality control method of CT system couch tops for treatment planning via an advanced 3D coordinate measuring machine
Source: J Appl Clin Med Phys. 2025 Dec 10;26(12):e70409. doi: 10.1002/acm2.70409 (PMC12695700; doi:10.1002/acm2.70409)
Supplement: Supplementary file 1 — Supporting information [file ACM2-26-e70409-s001.docx]

# Supplementary Material

# Verification of Measurement Accuracy in a CT Room

## Verification Method

The measurement accuracy of 3D-CMMs reportedly deteriorates under the influence of environmental factors, such as temperature, humidity, and air currents from air conditioning. ^1-4^ If the near-infrared light emitted by the markers attached to the probe interferes with the wall laser beams in a CT room, the intensity of the near-infrared light will change, which may affect the measurement accuracy. To verify the measurement accuracy of the 3D-CMM within a CT room, we conducted tests under conditions akin to clinical practice, taking into account environmental factors such as temperature, humidity, and air currents from air conditioning, as well as potential interference from wall laser beams. A three-axis linear stage with a micrometer capable of 0.01 mm increments and an accuracy of 0.03 mm was used. We mounted the stage on an index bar atop the CT couch top. The linear stage was then shifted 5 mm along each axis individually, followed by a simultaneous 5 mm shift along all axes because the couch top is expected to move in all three axes simultaneously. Measurements were performed three times before and after each shift, with the probe positioned in a predefined hole on the linear stage. The accuracy of the 3D-CMM was assessed by comparing the measured shifts to the known values of the linear stage shifts.

The uncertainty of the measurement depends on the distance between the camera unit and measurement point, L (mm), and is calculated using the following equation ^5^:

Uncertainty = ± (25 + 5L/1000) μm. (1)

Measurements were performed at three positions to investigate this effect, with the index bar set to align with the wall laser position (Long = 0), 700 mm toward the head of the couch top (gantry side) from the wall laser position (Long = 700), and 1400 mm from the wall laser position (Long = 1400). For all measurements, the camera unit was located approximately 3000 mm from the wall laser position (Long = –3000) (Figure 3).

## Results

The differences between the applied 5 mm and measured shifts are listed in Table S1. The discrepancies signify the variance between the 5 mm shift executed by the linear stage and the shift detected by the 3D-CMM. The maximum difference was 0.08 mm, and the SD over three measurements was within 0.05 mm. No differences were observed in the measurement accuracy based on the shifted coordinate axes. The effect of the distance from the camera unit on the measurement accuracy was insignificant within the range of the CT room.

Table S1. Results of measurement accuracy verification at various measurement points in the CT room.

| Measurement point | | Shifted axis | Mean deviation^a^ ± one standard deviation | | |
| --- | --- | --- | --- | --- | --- |
|  |  |  | Long (mm) | Lat (mm) | Vrt (mm) |
| Long = 1400 | | Long | **0.04 ± 0.05** | –0.01 ± 0.00 | –0.01 ± 0.01 |
|  |  | Lat | –0.01 ± 0.00 | **–0.01 ± 0.00** | 0.00 ± 0.00 |
|  |  | Vrt | –0.02 ± 0.02 | –0.02 ± 0.00 | **0.03 ± 0.00** |
|  |  | All^b^ | **–0.08 ± 0.04** | **–0.04 ± 0.02** | **0.03 ± 0.02** |
| Long = 700 | | Long | **–0.03 ± 0.04** | 0.01 ± 0.02 | –0.02 ± 0.02 |
|  |  | Lat | 0.01 ± 0.01 | **–0.01 ± 0.01** | –0.01 ± 0.02 |
|  |  | Vrt | 0.02 ± 0.02 | 0.00 ± 0.01 | **–0.03 ± 0.01** |
|  |  | All^b^ | **0.06 ± 0.03** | **0.03 ± 0.02** | **–0.03 ± 0.01** |
| Long = 0 | | Long | **0.05 ± 0.04** | –0.01 ± 0.01 | 0.00 ± 0.00 |
|  |  | Lat | –0.02 ± 0.02 | **–0.04 ± 0.00** | 0.00 ± 0.00 |
|  |  | Vrt | –0.02 ± 0.02 | –0.01 ± 0.00 | **0.03 ± 0.00** |
|  |  | All^b^ | –0.01 ± 0.03 | 0.03 ± 0.02 | 0.05 ± 0.02 |

^a^ deviation = 5 mm shift via linear stage – detected shift via 3D-CMM; ^b^ All indicates that all axes have been shifted 5 mm simultaneously; the axis undergoing the shift is highlighted in bold font.

## Discussion

The Keyence wide-area 3D-CMM used in this study can perform highly accurate measurements under the environmental conditions of CT rooms (Table 1). The measurement accuracy of 3D-CMMs has been reported to be affected by environmental conditions such as temperature and humidity. ^1-3^ However, the specifications ^5^ for the Keyence wide-area 3D-CMM recommend an operating temperature of +10–35 °C and an operating humidity of 20%–80% RH, which is considered sufficient for the general environment in CT rooms. Furthermore, a previous study ^4^ reported that the deterioration in measurement accuracy due to air currents caused by air conditioners is only a few micrometers, and, in any case, this effect can be eliminated by turning off the air conditioner while performing QC. The measurement point Long = 0 was the wall laser position where the wall laser hit the probe the most strongly. Therefore, measurements were performed to determine the effect of the wall laser light. The wall laser installed in the CT room of our institution was a green semiconductor laser (wavelength: 515 nm) (ALPC-G; Takenaka Optonik Co., Ltd., Kyoto, Japan). No indication that the wall laser light, under the study conditions, was affecting the measurements was noted. Moreover, the wavelength of the near-infrared light emitted by seven markers attached to the probe was 870 nm, which deviated from the wavelength of the wall laser, suggesting that the effect was minimal. Similar to the effect of the air conditioner, this effect can be eliminated by turning off the laser while performing QC. Based on the above findings, the distance from the camera unit was determined to have the highest impact on the measurement accuracy. Measurements were performed at Long = 700 (imaging plane) and Long = 1400 to assess the effect of the distance from the camera unit. Using Eq. (1), the distances from the camera unit were obtained as 3700 and 4400 mm, resulting in a measurement uncertainty of ±0.019 (Long = 700) and ±0.022 mm (Long = 1400), respectively. Because the calculated measurement uncertainty was considerably small, its effect could not be detected during measurements. Therefore, the location of the camera unit was appropriate. Within the range of the displacement measurement of the couch top, the effect of the distance from the camera unit on the measurement accuracy is considered negligible. The maximum deviation was 0.08 mm. Considering that the accuracy of the micrometer was 0.03 mm, the Keyence wide-area 3D-CMM can be measured with submillimeter-level accuracy in CT rooms.

## References

1. Harvie A. Factors affecting component measurement on coordinate measuring machines. *Precis Eng*. 1986;8(1):13-18.
2. Vo TA, Dung TH, Kim HC. Design of a High Accuracy 3-Axis Coordinate Measuring Machine Working on the Shop Floor. *Adv. Mater. Res*. 2015;1125:521-525.
3. Muñoz PP, García JAA, Mazo JS. Analysis of the initial thermal stabilization and air turbulences effects on Laser Tracker measurements. *J Manuf Syst*. 2016;41:277-286.
4. Sudatham W, Matsumoto H, Takahashi S, Takamatsu K. Diagonal in space of coordinate measuring machine verification using an optical-comb pulsed interferometer with a ball-lens target. *Precis Eng*. 2016;43:486-492.
5. Keyence Corporation. Wide-area three-dimensional coordinate measuring machine WM series user’s manual; 2022
